# Assessing the ecological impact of banana farms on water quality using aquatic macroinvertebrate community composition

**DOI:** 10.1007/s11356-016-8248-y

**Published:** 2017-01-23

**Authors:** Ola Svensson, Angelina Sanderson Bellamy, Paul J. Van den Brink, Michael Tedengren, Jonas S. Gunnarsson

**Affiliations:** 1 Department of Ecology, Environment and Plant Sciences (DEEP), Stockholm University, S-10961 Stockholm, SE Sweden; 2 Sustainable Places Research Institute, Cardiff University, 33 Park Place, Cardiff, CF10 3BA UK; 3 Department of Aquatic Ecology and Water Quality Management, Wageningen University, P.O. Box 47, 6700 AA Wageningen, Wageningen, The Netherlands; 4 Alterra, Wageningen University and Research, P.O. Box 47, 6700 AA Wageningen, Wageningen, The Netherlands

**Keywords:** Costa Rica, Banana production, Benthic macroinvertebrates, Water quality, Monitoring, Risk assessment

## Abstract

In Costa Rica, considerable effort goes to conservation and protection of biodiversity, while at the same time agricultural pesticide use is among the highest in the world. Several protected areas, some being wetlands or marine reserves, are situated downstream large-scale banana farms, with an average of 57 pesticide applications per year. The banana industry is increasingly aware of the need to reduce their negative environmental impact, but few ecological field studies have been made to evaluate the efficiency of proposed mitigation strategies. This study compared the composition of benthic macroinvertebrate communities up- and downstream effluent water from banana farms in order to assess whether benthic invertebrate community structure can be used to detect environmental impact of banana farming, and thereby usable to assess improvements in management practises. Aquatic invertebrate samples were collected at 13 sites, using kick-net sampling, both up- and downstream banana farms in fast flowing streams in the Caribbean zone of Costa Rica. In total, 2888 invertebrate specimens were collected, belonging to 15 orders and 48 families or taxa. The change in community composition was analysed using multivariate statistics. Additionally, a biodiversity index and the Biological Monitoring Working Party (BMWP) score system was applied along with a number of community composition descriptors. Multivariate analyses indicated that surface waters immediately up- and downstream large-scale banana farms have different macroinvertebrate community compositions with the most evident differences being higher dominance by a single taxa and a much higher total abundance, mostly of that same taxon. Assessment of macroinvertebrate community composition thus appears to be a viable approach to detect negative impact from chemical-intensive agriculture and could become an effective means to monitor the efficacy of changes/proposed improvements in farming practises in Costa Rica and similar systems.

**Electronic supplementary material:**

The online version of this article (doi:10.1007/s11356-016-8248-y) contains supplementary material, which is available to authorized users.

## Introduction

Costa Rica is one of the richest countries in the world in terms of biodiversity and considerable effort goes to conservation and protection. Several protected areas, some being wetlands or marine reserves, are however, situated downstream agricultural areas, where the use of agrochemicals is very high (Schreinemachers and Tipraqsa 2012) and run-off into nearby surface waters is of particular concern (Castillo et al. 2006). A major contributor of agrochemicals to the surrounding environment is the large-scale banana production, which receives an average of 57.5 pesticide applications per year as well as 2775 kg/ha of synthetic fertilizers (Bellamy 2013; Bravo et al. 2013). Several of the pesticides used in banana production have been detected in the aquatic environment downstream of banana production areas (Castillo et al. 2006), some in concentrations expected to have acute or chronic toxic effects on aquatic organisms according to toxicity values derived from laboratory toxicity tests (Diepens et al. 2014; Arias-Andrés et al. 2016; Rämö et al. 2016).

Banana companies are today increasingly aware of the need to reduce their negative environmental impact, and several changes in management practises have resulted in some companies being certified according to one of several certification systems (e.g. Rainforest Alliance and ISO14000). Attempts to reduce environmental impact by farms include the following: sediment traps that are constructed to reduce erosion and capture/retain pesticides adhered to solids; riparian vegetation zones that are planted/left to intercept spray drift, prevent erosion and reduce surface flow and leaching of pesticides; manual chopping of weeds instead of using herbicides; manual injections of nematicides into the banana plant instead of applying soil granular nematicides; and post-harvest applications of fungicides using brushes instead of fumigation chambers, thereby reducing the amount of pesticides used.

Some of these practises may reduce the negative impact on the environment, but few ecological field studies have been done to evaluate the efficiency of mitigation strategies that aim to reduce negative environmental impact in Costa Rican rivers and in similar tropical aquatic systems. Monitoring changes of benthic macroinvertebrate community composition is commonly used in monitoring programs and ecological status assessments of freshwater and marine coastal systems around the world (e.g. van Hoey et al. 2010; von der Ohe and Goedkoop 2013). In this study, we evaluate changes in benthic community composition up- and downstream from banana plantations as a means to evaluate ecological effects of current agricultural practises and the efficiency of proposed improvements, as well as a complement to chemical analysis of pesticide residues in environmental risk assessment.

Benthic macroinvertebrates are usually abundant in rivers, represent several trophic levels, participate in nutrient cycling and differ in sensitivity to pollution. Most of them have small home ranges, at least in aquatic stages, and usually have long life cycles and thus are good bioindicators as they provide information about the water quality integrated over a longer time period, compared to the values given by water samples taken at discrete points in time. Pesticide and nutrient levels in the aquatic environment can be expected to vary, with peaks after application and high rainfall events. Monitoring of pesticide levels thus requires a very frequent sampling to detect peak concentrations (Liess et al. 2003). Another concern is that toxic effects can result from exposure near or below the analytical detection limit for a given pesticide (Walter et al. 2002) or from a combination of pesticides and other stressors, e.g. temperature or high nutrient loads (Polidoro and Morra 2016). It is also important to consider the effect of chronic exposure to pesticides as well as the exposure to mixtures of several pesticides, which together can cause toxic effects through additive toxicity (Verbruggen and van den Brink 2010).

The possible additive or synergistic effects between stressors are a major concern in rivers, which receive irrigation and run-off water from banana farms. Large-scale banana farming relies on the use of fungicides, nematicides, insecticides and herbicides. Most often, several different compounds of each type of pesticide are applied over the year in order to minimize risk of inducing resistance in pests. The extensive system of drainage canals in a typical banana farm causes increased stream flashiness and sedimentation and due to high precipitation a substantial amount of pesticides and nutrients end up in the aquatic environment. Non-target aquatic organisms further downstream will thus be subjected to a complex mixture of toxic substances, fertilizers and changes in stream flow. To assess cumulative effects of several physical and chemical stressors, responses thus have to be studied at the community or ecosystem level and using benthic macroinvertebrates has proved to be a cost-effective monitoring tool.

In the present study, we evaluated whether the overall impact of banana farming affects aquatic benthic macroinvertebrate fauna in waters subjected to agricultural run-off to test if benthic macroinvertebrate community composition can be used as a bioindicator of ecological stress to these aquatic ecosystems. Our research hypothesis was that surface waters downstream of banana farms will have a different benthic macrofauna community composition with a lower diversity compared to upstream sites. The changes in community composition were assessed at the family level, with the objective of testing a robust, ecologically relevant method to detect environmental impact of agricultural run-off.

## Material and methods

### Sites

**Fig. 1:**
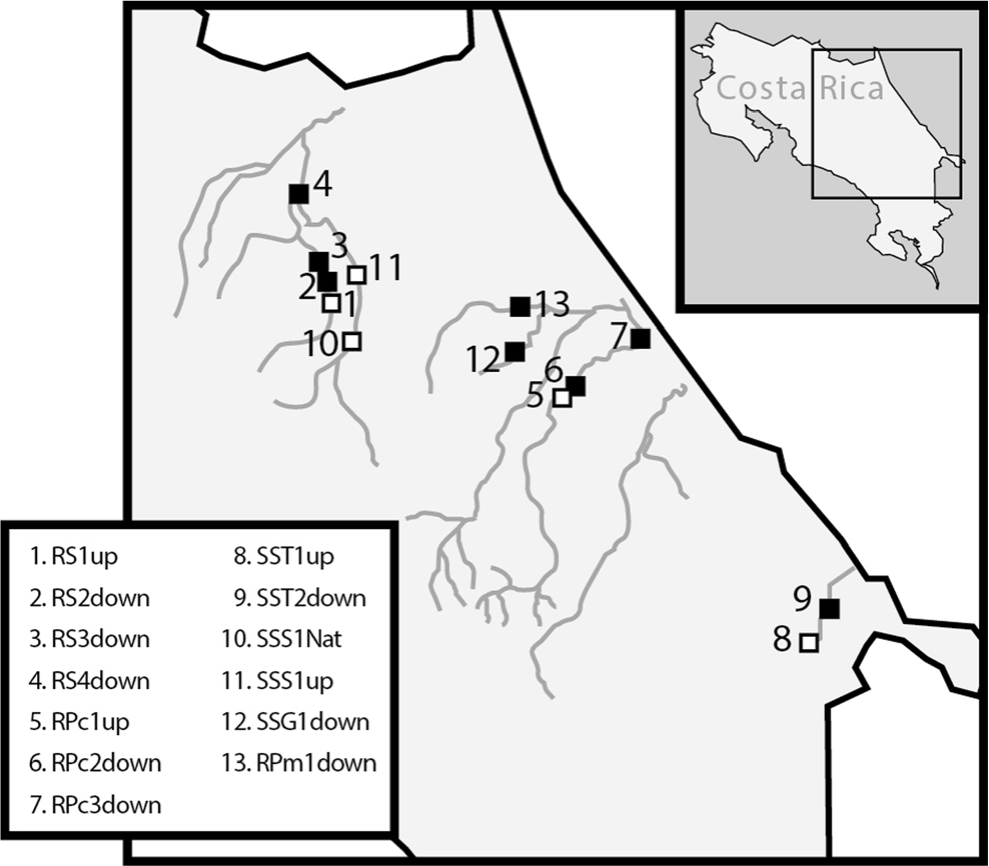
Map of Costa Rica, showing the location of the 13 sampling sites and rivers sampled. For GPS coordinates and description of sites and farming type at each site see Table 1. For number of composite samples and mean values for different community structure descriptors see Table 2.*White* and *black squares* denote upstream and downstream sites respectively. *RS* Río Sucio, *RPc* Río Pacuare, *SST* Small stream in Talamanca, *SSS* Small stream in Sarapiquí, *SSG* Small stream in Guácimo and *RPm* Río Parismina.

Aquatic benthic invertebrate samples were collected at 13 sites in the Caribbean low-lands of Costa Rica between March 8 and April 26, i.e. during the dry season, 2007 (Table 1). Sites were chosen both up- and downstream in rivers and watercourses receiving run-off from banana farms and at sites assumed not to be affected by banana farming (Fig. 1). A high natural variability in community composition can be expected between and along streams. Both stream order and stream size influence taxa richness and community structure (Malmqvist and Hoffsten 2000; Vannote et al. 1980), as do local factors, such as riparian characteristics, water chemistry and in-stream habitat structure. Sampling sites were thus, when possible, chosen in pairs along the same watercourse, with one site situated upstream and the second downstream banana farms (Fig. 1). By comparing sites in an upstream-downstream fashion, the difficulty with interpretation associated with the natural inter-stream variation is greatly reduced. Spatial habitat heterogeneity, current velocity at base and high flows, and type of substrate also affect within-site diversity of stream invertebrates (Beisel et al. 2000). Sampling of highly similar habitats was therefore favoured to reduce this variability, with fast flowing streams, mostly cobbles for substrate in runs and riffles, and no or little macrophytes being the preference (see Table 1 for comparison between sites). Keeping to those prerequisites in combination with limited access, only three rivers were sampled in a true, replicated upstream-downstream fashion (see Table 2).

**Table 1:**
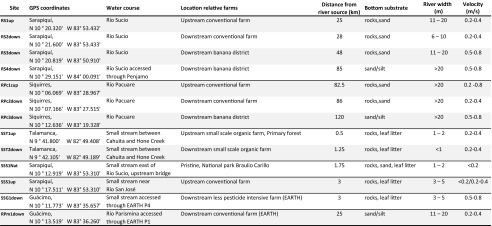
Sampling sites with GPS coordinates, a description of the site and some characteristics including the distance from the source of the surface water, the type of substrate, the width of the river and the velocity of water flow at the sampling site

**Table 2:**
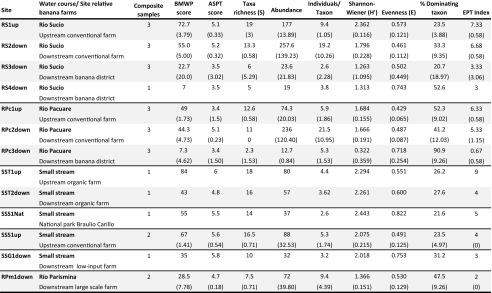
Benthic community structure comparisons of upstream and downstream sites with mean values (standard deviation within brackets). Each composite sample consists of six pooled kick-samples, corresponding to approximately 1 m^2^

In the provinces of Sarapiquí and Siquirres, where conventional, large-scale banana farms are abundant, Río Sucio and Río Pacuare were sampled up- and downstream of single farms as well as downstream ‘banana districts’, with large-scale banana farms being the dominating land use (Fig. 1, Table 1). In addition, samples were taken in two small streams, with one site (SSS1Nat) located within Braulio Carrillo National Park near the source of Río Sucio, and another site upstream from banana farms (SSS1up). In the province of Talamanca, a small stream between Cahuita and Hone Creek was sampled up- and downstream a small-scale, certified organic farm. Finally, in the province of Guácimo a small, first order stream located adjacent to a low-input banana farm within EARTH University a few hundred meters west of Río Dos Novillos was sampled, as was Río Parismina downstream a conventional banana farm. At each site, the following data were recorded in a field protocol: GPS-position, date and time, rainfall within last 24 h, present cloud conditions, measurements of temperature (air and water), assessment of river width, depth, current, velocity, turbidity, colour and amount of shade, assessment of substrate composition (amount boulders, stones, sand, silt, plant material etc.). Additionally, we measured approximate distances from the sample site to the source of the stream or river sampled.

### Sampling and identification of macroinvertebrates

We used kick net sampling since Armitage (1978) and Pollard (1981) found the method to give consistent results. By disturbing the bottom, specimens are dislodged and drift into a net held immediately downstream. The net used was a D-framed 40-cm-wide kick net with 0.5 mm mesh size. To obtain a representative composite sample, an area equivalent to the area of the net was disturbed at six sub-sampling positions (randomly chosen within an area of about 25 m^2^). Each sampling position was approached either at a right angle to the flow direction or from downstream in order not to sample where the bottom had been inadvertently disturbed. The net was held as close as possible to the streambed. The substrate in front of the net was disturbed, either by kicking or by hand. The latter was favoured due to the substrate; in most cases rocks of a size that would not easily turn over by kicking. Animals and epiphytes were dislodged by brushing hands over rock surfaces and collected with the softer substrate into the net. All sweeping of substrate/disruption of bottom was directed toward the net to reduce loss of swimming specimens. One composite sample represents approximately 1 m^2^ and up to three composite samples were collected at each site. Sites are designated by an abbreviation for actual watercourse, a numeral for relative position along the watercourse and whether it is an up- or downstream site.

Samples were transferred to labelled containers and preserved in 70% alcohol. Sorting and identification was done to family level (Oligochaeta, Acarina were only identified to order and Bivalvia to class) under stereoscope using relevant taxonomical keys (Thorp and Covich 1991; Roldán Pérez 1992). Reference specimens were deposited at Costa Rica’s National Institute of Biodiversity (INBio).

### Analyses of benthic community composition

Our research question was whether surface waters downstream banana farms have a different benthic macroinvertebrate community composition with a lower diversity compared to surface waters upstream banana farms. The effect of agricultural run-off on benthic macroinvertebrate community composition was studied with multivariate statistics. Similarity between invertebrate communities up- and downstream plantations was assessed using principal component analysis (PCA) (Van den Brink et al. 2003; van Wijngaarden et al. 1995) using CANOCO (version 5) (Ter Braak and Smilauer 2012). PCA was used since the invertebrate data set had a short length of gradient (2.8 SD; van Wijngaarden et al. 1995), while the abundance data were Ln (2× + 1) transformed (see Van den Brink et al. 2000 for rationale). Analyses were performed on mean values per site where more than one composite sample exists. PCA generates an ordination diagram, which allows comparison of how closely the different sites are related to each other in terms of community composition and additionally show how the taxa composition varies between sites, i.e. upstream or downstream banana farms. Sites that lie close together on the PCA diagram share a more similar community composition than those sites that lie further apart (Ter Braak 1995). Site characteristics were introduced as supplementary explanatory variables to assess the correlations between taxa abundance values and the levels of the explanatory variables (Van den Brink et al. 2003).

The Biological Monitoring Working Party (BMWP) score system (National Water Council 1981), originally developed in Great Britain as a rapid and sensitive method to determine water quality using macroinvertebrate sensitivity to organic pollution has also been adapted for use in tropical environments, and has proved to correctly assess water quality in e.g. Thailand (Mustow 2002). The BMWP scores adapted to Costa Rican conditions according to Springer et al. (2007) were used to rank the sites based on their indicated sensitivity to organic pollution. In the BMWP system, a score (from 1 for the most tolerant to 10 for the most sensitive) is assigned to different taxa depending on their sensitivity to organic pollution and only requires identification to the family level (Oligochaeta only to class). In order to make interpretation less sensitive to sampling effort, Armitage et al. (1983) and others (e.g. Friedrich et al. 1996) suggest dividing the total sample score with the number of contributing taxa, giving the result as Average Score Per Taxon (ASPT). These values as well as number of families, individuals per taxon, Shannon-Wiener diversity index, EPT index (Lenat 1988) and percent contribution of the most abundant taxon out of the total abundance were compared to determine if differences between sites could be detected.

## Results

**Fig. 2:**
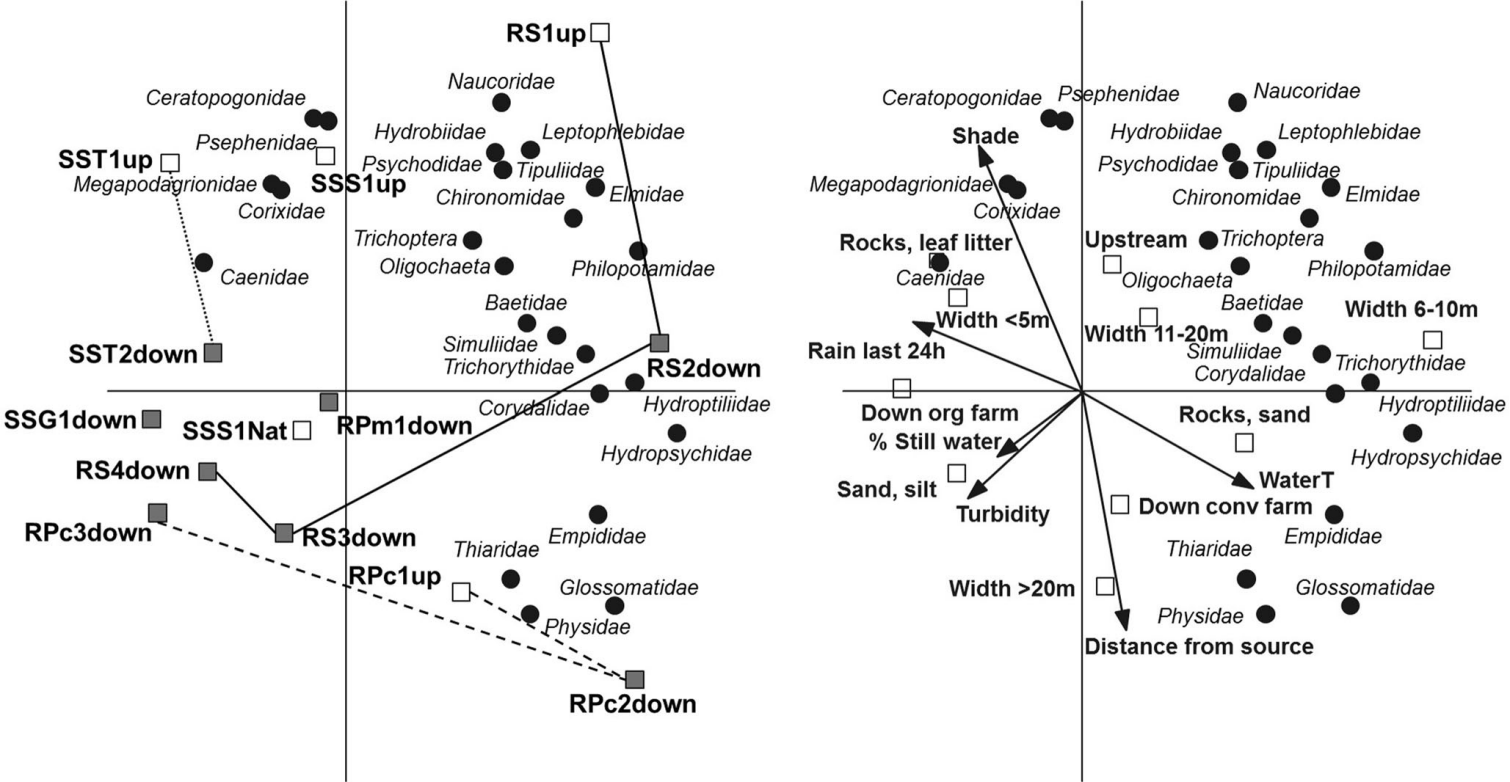
PCA biplots showing the variation in taxa composition between the sites (Fig. 2a) and the correlation between the taxa and the measured explanatory variables (Fig. 2b) Of the variation in taxa composition, 30% is displayed on the *horizontal axis* and another 18% on the *vertical axis*. Analyses were performed on mean values where more than 1 composite sample was taken

In total, 2888 specimens were collected, belonging to 15 orders and 48 families or taxa. The PCA diagram clearly shows the differences in community composition between the rivers and between up- and downstream sites (Fig. 2). The horizontal and vertical axes (displaying 30 and 18% of the total variation, respectively) show that most upstream sites and taxa are located in the upper, right part of the diagram, while most downstream sites are located in the lower, left quadrant, where also no taxa are located. This shows that most downstream sites have a poorer community composition compared to the upstream sites. Surprisingly, the sample taken in the national park (Sarapiquí) clusters together with the downstream sites. The samples taken in the Río Sucio and Río Pacuare just below the conventional farm are still relatively rich in taxa (i.e. located on the right side of the diagram), while their more downstream located sites are located in the lower, left quadrant.

Figure 2B shows how the community composition at each site relates to the explanatory variables. The sites located in the left, lower part of the diagram, have a low number of taxa and are correlated with being downstream both organic and conventional farms, high turbidity, a high % of still water, a high presence of sandy, silty substratum, a high distance from source and a large width of the river. Upstream sites are correlated with a higher amount of shade, an intermediate river width and a higher number of taxa.

The number of families per site ranged from 2.3 to 19 with a mean value of 11.6 taxa per site. Family/taxa richness was higher in upstream samples as were BMWP-scores and Shannon-Wiener diversity values (Table 2). In the Talamanca stream passing an organic farm, there were fewer orders present in the upstream site (site SST1up, 7 orders) compared to the downstream site (site SST2down, 11 orders). Only 6 orders were found at site SSS1Nat within the national park, but 14 families, meaning higher within-order diversity (Table 2). With regards to abundance, Chironomidae (i.e. midge larvae) was the most abundant family at several sites (RS1up, RS2down, SST2down, SSS1Nat, RS4down and RPc3down) and were found at all sites. The range of Chironomid abundance varied between 10% downstream a banana district along Río Sucio (site RS3down) to 91% downstream another banana district along Río Pacuare (site RPc3down). Within the order of Trichoptera (caddisflies), Glossomatidae, Hydropsychidae and Philopotamidae all had higher abundances at downstream sites despite high BMWP scores (score 8, 5, 7, respectively). Leptoceridae, on the other hand, were fewer downstream (score 8; Table 2). EPT index values varied between 0.63 downstream a banana district and 9 upstream an organic farm, and were higher at upstream sites.

Invertebrate diversity at the order level varied between 3 and 11 orders (mean 7.1), and was higher in upstream samples except for sites SST1up and SST2down (up- and downstream the organic farm). Diptera were found at all sites (total 624 individuals), and were the dominant order at site RS4down and RPc3down, both downstream banana districts. At site RS4down, one Diptera family contributed with 53% of total abundance while at site RPc3down two families added up to 92%. This is in contrast to site SSS1Nat, in the national park, where Diptera contributed 32% of the sample, but were represented by four families. Trichoptera contributed a total of 1185 individuals. Ephemeroptera (553 individuals), missing only at site RPc3down, were the dominant order at site RS3down, both sites situated downstream large banana districts. Coleoptera were missing at sites RS3down, RS4down, and RPc3down, i.e. the sites downstream large banana districts. Plecoptera were found only at site SST1up and SSG1down, i.e. upstream the organic and downstream the low-input farm. Only a few taxa were absent at most sites. None of those taxa present only at one single site were found downstream large-scale conventional banana farms. Special note should be taken that oligochaetes (with the lowest sensitivity score, 1) were only found at upstream sites.

## Discussion

The aim of the study was to assess if benthic macroinvertebrate community composition at the resolution of family level could be used to evaluate improvements in banana farming practises. The PCA ordination plots show differences in community composition between rivers but community composition also differed between up- and downstream sites in the same river (Fig. 2). The explanatory variable ‘upstream’ was positively associated with a larger number of taxa (Fig. 2), indicating a general trend that upstream sites are more species-diverse. Higher river width was inversely correlated with number of taxa indicating that other factors than oxygen stress are affecting these communities. The fact that Oligochaetes, normally very tolerant to low oxygen levels, are only found at upstream sites further supports this conclusion (Fig 2). Oligochaetes have been found to be relatively sensitive to fungicides (Cuppen et al. 2000), which have been found in surface waters downstream banana farms in streams nearby (Castillo et al. 2000; Diepens et al. 2014; Arias-Andrés et al. 2016; Echeverría-Sáenz et al. 2016; Rämö et al. 2016). Thus, pesticides used by banana farms may be influencing patterns shown in the biplots presented here (Fig. 2).

The BMWP scores, taxa richness and diversity indices were slightly lower downstream conventional farms than upstream, interpreted as a response to water quality or habitat deterioration. It should be emphasized that upstream ‘reference’ sites in some cases are affected by land use further upstream, i.e. not to be considered to represent pristine conditions (Fig. 1). This is e.g. the case for sites RS1up and RPc1up, the upstream conventional farm sites in the pairwise comparison. Therefore, a reduction of taxa richness and loss of the most sensitive species may have already occurred further upstream, possibly explaining the sometimes modest differences found when sites up- and downstream banana farms were compared (Table 2). The small difference in taxa richness is to some extent related to an increased richness of tolerant taxa at some of the downstream sites. Environmental impact is therefore difficult to interpret from taxa richness alone. Diversity index figures are likewise unaffected if one taxon replaces another in response to pollution, and, accordingly, differences in diversity were moderate. A metric that showed a distinct difference was abundance, where, contrary to results by Paaby et al. (1998), abundance was found to be higher at sites downstream conventional farms. In the streams not affected by banana farms and the one passing a small-scale organic farm the number of individuals per taxon was quite low, but the number of individuals per taxon almost doubles downstream large-scale banana farms, despite the short distance. Higher abundance in these cases co-varies with increased dominance by one or two taxa (Hydropsychidae and Chironomidae at site RS2down resp. Glossomatidae at site RPc2down), indicating stress (Pearson and Rosenberg 1978) and evenness have been found to respond faster than species richness to environmental stress (Chapin et al. 2000). Previous as well as recent studies in nearby rivers, which also receive fertilizers and pesticides from banana plantations have shown similar effects on the invertebrate community (Castillo et al. 2006; Pringle and Ramirez 1998; Echeverría-Sáenz et al. 2016). The ASPT (Average Score Per Taxon) score, contrary to expected, suggested that there were more sensitive taxa downstream banana farms compared to sites upstream (Table 2). Oligochaetes, with a low score, were present only at the upstream sites possibly due to fungicide exposure, lowering the ASPT scores compared to downstream sites and this highlights the shortcomings of many water quality score systems. The BMWP score system is based on sensitivity of families to oxygen depletion, which is used as an indicator of organically polluted surface waters. While this generally correlates with agricultural runoff, particularly with regards to sediment and fertilizer run-off, the BMWP score system does not reflect species’ sensitivity to pesticide exposure. Rico and Van den Brink (2015) propose a trait-based methodology using focal species that also incorporates landscape characteristics to improve insecticide risk assessment based on invertebrate monitoring. However, at the community level, the response of aquatic invertebrates to stress, e.g. lower diversity and higher abundance of some more tolerant species can be expected to be similar regardless of the stressor (e.g. oxygen deficiency or contaminants such as metals, oil or polycyclic aromatic hydrocarbons (Diaz 1992)).

Pringle and Ramirez (1998) found Diptera (e.g. Chironomidae) and Ephemeroptera to be dominant insect groups at sites both in primary forest and in streams draining banana plantations. Sensitivity varies among Dipteran families, but Chironomidae, according to the BMWP score system, are considered tolerant to organic pollution and oxygen deficiency. In the present study, Chironomidae were found at all sites, and were also to varying degree the dominating taxon at six of the sites, four of which were downstream sites. At the site furthest downstream Río Pacuare (site RPc3down), receiving run-off from several large-scale banana farms, the abundance of Chironomidae was one order of magnitude greater than that of the second most abundant taxon, and the extremely low total abundance at this site indicates very poor conditions. Ephemeroptera, generally considered as relatively sensitive to organic pollution, were not represented at the aforementioned site, but dominated at four other sites, two of which were situated downstream banana farms. However, the Ephemeroptera families observed in these streams include several species (*Baetis spp*. and *Caenis spp.*) that are commonly found in organically enriched streams (Barbour et al. 1999). Thus, it is important to consider that while scores are based on aggregate sensitivity of species within the order or family, there may be some species more or less sensitive than the aggregate score given to a group. Other examples from the data are Leptophlebidae (Ephemeroptera) and Glossomatidae (Trichoptera), two families with high BMWP scores, which were also present in high numbers or even dominant at downstream sites.

Sampling of rivers affected by small-scale organic farms proved to be difficult due to the lack of permanent streams or due to streams being impacted by other land use upstream. The one sampled, though, presented an upstream reference site within primary forest, and the banana farm being the only land use. The choice of sampling sites was in general limited by access difficulties in combination with aforementioned requisites of comparable habitats. Ideally more sites would have been sampled and preferably some with large-scale banana farms being the only land use. However, with the upstream-downstream samples taken within a rather short distance, and banana farming being the most significant land use and also the most chemical intense, one can assume that most of the observed effects are due to production practises on banana farms.

## Conclusions

Although it can be difficult to distinguish natural variations in e.g. diversity and community composition from the effects of human impact, the consistent pattern with increasing dominance by a single or only a few taxa at downstream sites indicates an impact from banana farming. The present study hints at a lower impact by organic farming, but lacks replication to support it. The fact that differences, although small, were detected when comparing up- and downstream single farms, implies that monitoring of macroinvertebrate community composition is useful for assessing management practises and improvements proposed or introduced by the banana industry aiming to produce bananas in a more sustainable way. As monitoring invertebrate community composition is highly ecologically relevant, we recommend that it should be done in combination with chemical analysis of pesticide residues in environmental monitoring programs in Costa Rica and in ecological risk assessment of these rivers and similar aquatic systems.

## Electronic supplementary material

ESM 1 (XLSX 11 kb).
